# Whole lung radiomic features are associated with overall survival in patients with locally advanced non-small cell lung cancer treated with definitive radiotherapy

**DOI:** 10.1186/s13014-025-02583-1

**Published:** 2025-01-17

**Authors:** Meng Yan, Zhen Zhang, Jia Tian, Jiaqi Yu, Andre Dekker, Dirk de Ruysscher, Leonard Wee, Lujun Zhao

**Affiliations:** 1 Department of Radiation Oncology, Key Laboratory of Cancer Prevention and Therapy, Tianjin Medical University Cancer Institute & Hospital, National Clinical Research Center for Cancer, Tianjin’s Clinical Research Center for Cancer, Tianjin, 300060 China; 2 Zhejiang Cancer Hospital, Hangzhou Institute of Medicine (HIM), Chinese Academy of Sciences, Hangzhou, Zhejiang 310022 China; 3 Department of Radiation Oncology (Maastro), GROW Research Institute for Oncology and Reproduction, Maastricht University Medical Centre+, Maastricht, The Netherlands; 4 Department of Radiation Oncology, National Cancer Center/National Clinical Research Center for Cancer/Cancer Hospital, Chinese Academy of Medical Sciences and Peking Union Medical College, Beijing, 100021 China

**Keywords:** Radiomics, Radiotherapy, Lung cancer, Overall survival

## Abstract

**Background:**

Several studies have suggested that lung tissue heterogeneity is associated with overall survival (OS) in lung cancer. However, the quantitative relationship between the two remains unknown. The purpose of this study is to investigate the prognostic value of whole lung-based and tumor-based radiomics for OS in LA-NSCLC treated with definitive radiotherapy.

**Methods:**

A total of 661 patients with LA-NSCLC treated with definitive radiotherapy in combination with chemotherapy were enrolled in this study, with 292 patients in the training set, 57 patients from the same hospital from January to December 2017 as an independent test set (test-set-1), 83 patients from a multi-institutional prospective clinical trial data set (RTOG0617) as test-set-2, and 229 patients from a Dutch radiotherapy center as test-set-3. Tumor-based radiomic features and whole lung-based radiomic features were extracted from primary tumor and whole lungs (excluding the primary tumor) delineations in planning CT images. Feature selection of radiomic features was done by the least absolute shrinkage (LASSO) method embedded with a Cox proportional hazards (CPH) model with 5-fold cross-internal validation, with 1000 bootstrap samples. Radiomics prognostic scores (RS) were calculated by CPH regression based on selected features. Three models based on a tumor RS, and a lung RS separately and their combinations were constructed. The Harrell concordance index (C-index) and calibration curves were used to evaluate the discrimination and calibration performance. Patients were stratified into high and low risk groups based on median RS, and a log-rank test was performed.

**Results:**

The discrimination ability of lung- and tumor-based radiomics model was similar in terms of C-index, 0.69 vs. 0.68 in training set, 0.68 vs. 0.66 in test-set-1, 0.61 vs. 0.62 in test-set-2, 0.65 vs. 0.64 in test-set-3. The combination of tumor- and lung-based radiomics model performed best, with C-index of 0.71 in training set, 0.70 in test-set-1, 0.69 in test-set-2, and 0.68 in test-set-3. The calibration curve showed good agreement between predicted values and actual values. Patients were well stratified in training set, test-set-1 and test-set-3. In test-set-2, it was only whole lung-based RS that could stratify patients well and tumor-based RS performed bad.

**Conclusion:**

Lung- and tumor-based radiomic features have the power to predict OS in LA-NSCLC. The combination of tumor- and lung-based radiomic features can achieve optimal performance.

**Supplementary Information:**

The online version contains supplementary material available at 10.1186/s13014-025-02583-1.

## Introduction

Identification of biomarkers to predict treatment outcomes is essential to achieve personalized treatment. Several studies have demonstrated that radiomics extracted from LA-NSCLC (locally advanced non–small-cell lung cancer) can predict prognosis, such as locoregional failure [1], distant metastasis [2], progression-free survival [3] and overall survival (OS) [4]. Throughout these studies reported in literature, the predictors/models were built based exclusively on tumor features. However, prognosis of NSCLC might not only depend on the tumor but also on the tumor micro-environment and on the host, and thus the interplay between these factors may have a significant role in treatment and prognosis [5–8]. Different cells derived from different organs and tissues may contribute to the heterogeneity of the tumor environment, which is thus related to prognosis of NSCLC [9]. In clinical studies, survival of NSCLC patients are associated with both intrinsic tumor aggressiveness and the environment into which the tumor grows [10]. Several studies have found that coexisting chronic obstructive pulmonary diseases (COPD) was associated with worse survival in patients with NSCLC [11–14] and coexistence of interstitial lung disease and NSCLC was also independent risk factors for shorter survival of NSCLC [15–18]. These studies suggest that the heterogeneity of lung tissue is associated with OS of NSCLC. Shuo W et al. [19] established a fully automated artificial intelligence system to mine whole-lung information from CT images to predict EGFR genotype and prognosis with EGFR-TKI treatment, and its performance was overall better than tumor-only based deep learning methods. This study demonstrated the potential of artificial intelligence to decode the lung tissue phenotype. Based on these studies, we hypothesized that whole-lung radiomic features may be correlated with OS of LA-NSCLC.

In this study, we extracted radiomic features of tumor and lung regions from the planning CT images of LA-NSCLC patients treated with definitive radiotherapy in combination with chemotherapy and developed OS prediction models. The predictive value based on tumor and lung tissue alone as well as the combination of both was investigated. Three independent validation sets with a large heterogeneity of patient were used to validate the performance of the proposed models. In addition, patients were stratified into different risk groups based on the radiomic score to assess the performance of the models.

## Methods

### Patients and data sets

**Fig. 1:**
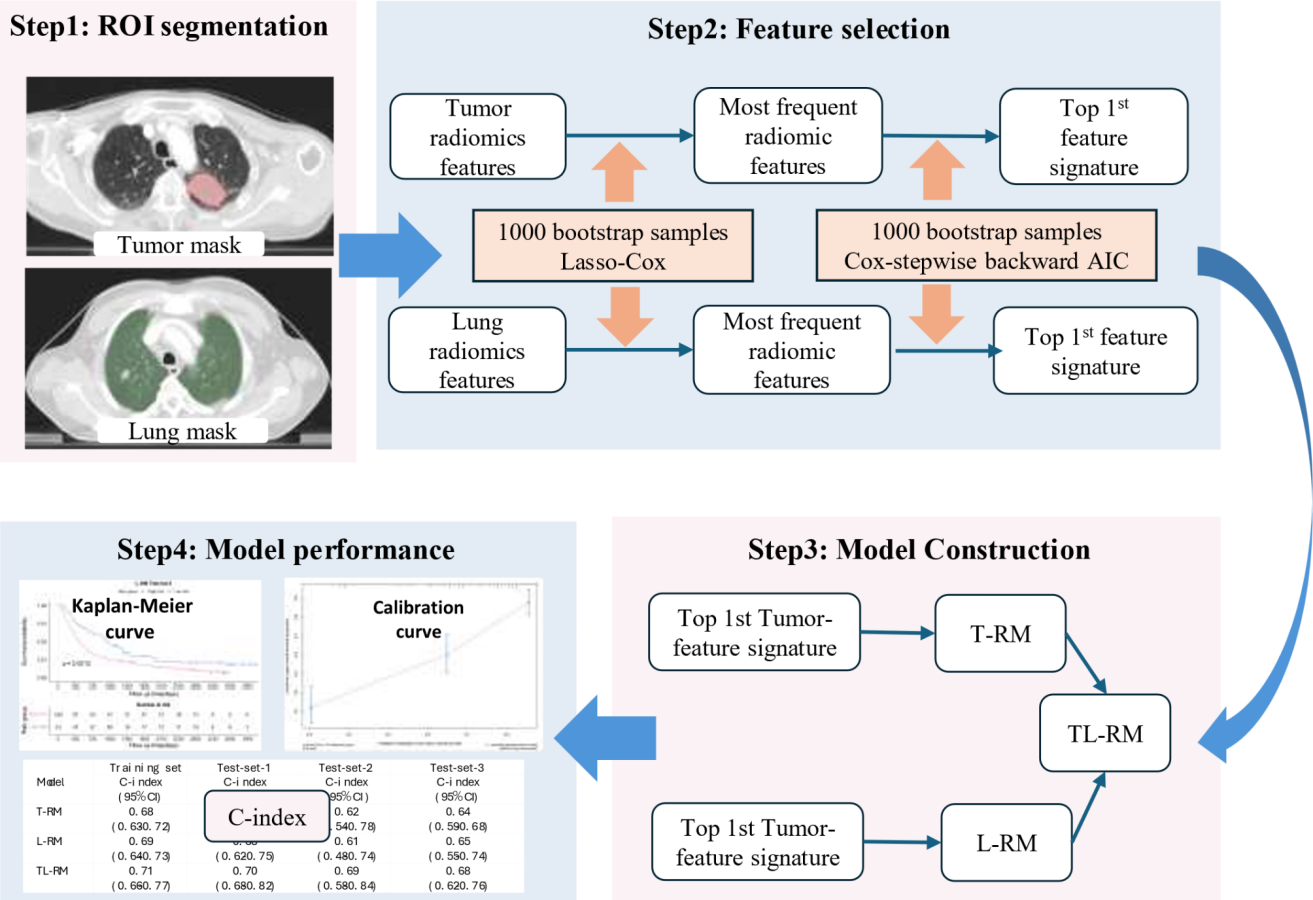
Workflow of the project. ROI segmentation: tumor region and whole-lung region segmented; Feature selection: 1000 unique bootstrap samples taken from all samples, features selected by correlation, least absolute shrinkage embedded with Cox proportional hazards model and Akaike information criterion for modeling; Model construction: tumor radiomics signature and lung radiomics signature for model construction; Model performance: model performance evaluated using discrimination, calibration and Kaplan-Meier analysis. Abbreviations: ROI: region of interest; Lasso-Cox: least absolute shrinkage embedded with Cox proportional hazards model; AIC: Akaike information criterion; T-RM: Cox proportional hazards model based on tumor signature; L-RM: Cox proportional hazards model based on lung signature; TL-RM: Cox proportional hazards model based on tumor signature and lung signature; C-index: Harrell concordance index

This retrospective observational study is a Transparent Reporting of a multivariate prediction model for Individual Prognosis Or Diagnosis (TRIPOD) type 3 investigation. This study was approved by our institution Ethics Committee (IRB/bc2021135) and confirmation of informed consent by patients was waived. The workflow of the study is shown in Fig. 1. We retrieved 349 LA-NSCLC patients who underwent definitive radiotherapy in combination with chemotherapy from January 2015 to June 2020 in Tianjin Medical University Cancer Institute and Hospital, in which patients from January 2015 to December 2016 and January 2018 to June 2020 served as training set (292 patients) and patients from January to December 2017 formed the independent test-set-1 (57 patients). More details on the criteria of inclusion and exclusion are given in Supplementary Materials A.

In addition, a multi-institutional prospective RTOG clinical trial dataset (RTOG0617 [20, 21]) (test-set-2) and a data set from a Dutch radiotherapy center - MAASTRO (test-set-3) [20, 22, 23] were used as external validation. They comprised of 83 patients and 229 patients, respectively. The patient (in external test sets) inclusion criteria is provided in Supplementary Fig. S1.

The endpoint of the study was OS, which is defined as the time from the date of the final radiotherapy fraction until death for any cause or lost follow-up (right censor).

### CT acquisition and ROI segmentation

All planning CT scans were acquired on a Brilliant (Philips Medical Systems; Best, The Netherlands) scanner in the training set and test-set-1. The scan parameters were set as follows: slice thickness 2–5 mm, tube voltage 120 kV, tube current 100 mAs, 512 × 512 pixels, vendor’s default convolution kernel was used for reconstruction. To reduce the influence of slice thickness on feature extraction, all planning CT images were isotopically resampled as 1 mm cubes with linear interpolation before segmentation of regions of interest (ROIs). Two ROIs (primary tumor and whole lung excluding primary tumor) were segmented. Before radiotherapy, a junior physician manually delineated the primary tumor and a senior radiation oncologist modified and reviewed this segmentation in the Pinnacle TPS (Philips Radiation Oncology Systems; Fitchburg, Wisconsin, United States), with image fusion against complementary imaging studies when possible (such as positron emission tomography). For the segmentation of lungs, in order to improve the efficiency and consistency of segmentation, a retrained deep-learning automatic contour model was used for lung segmentation based on a published model [24]. Then manual editing was performed by a radiation oncologists (author M.Y.), and the other experienced radiation oncologists (author Z.Z) independently reviewed the lung segmentations using 3D slicer software [25]. More details of the segmentation are available at Supplementary Material B.

### Radiomic features extraction and feature selection

In total 824 radiomic features were extracted based on Pyradiomics v3.7, covering 17 intensity histogram features, 14 shape features, 73 textural features and 711 wavelet radiomic features. More details about the radiomic features extraction are described in previous publications [26, 27]. The feature extraction parameters are given in Supplementary Materials C. As shown in the Fig. 1, two steps were adopted for selection of radiomic features. First, pair-wise feature correlation reduction; we used the R caret library to recursively reduce the total number of pair-wise Pearson correlation between any two features with Spearman correlation in excess of 0.9. Then the radiomic features were screened using least absolute shrinkage (LASSO) embedded with COX proportional hazards model (COX) with 5-fold cross-internal validation by the R glmnet package, with 1000 unique bootstrap samples from the whole training set. From each of the 1000 bootstraps, we ranked each individual feature according to how frequently the feature was retained by the LASSO-COX. Then, we selected a cut-off frequency for the top most frequently appearing individual features from the ranking table. Secondly, the radiomic features selected over the cut-off point were imported into the “feature pool” for further selection based on COX and stepwise backward Akaike information criterion (AIC) for the same 1000 bootstrap samples as in step 1.

Finally, the radiomic feature combination (signature) that ranked first in frequency was selected for model building. Prior to modeling, radiomic features were normalized by the Z-score. The same feature selection approach was used for tumor- and lung-based radiomics signatures.

### Construction of the models

The tumor radiomic signature-based COX regression model (T-RM) was developed and the tumor radiomic prognostic score (T-RPS) was calculated. The same approach was performed for the COX regression model based on lung signature (L-RM) and lung radiomics prognostic score (L-RPS). A combined tumor and lung-based model (TL-RM) was constructed using T-RPS and L-RPS as covariates. The prognostic score (TL-RPS) was calculated using a Cox regression model, with weights for the covariates determined by the model’s coefficients estimated during the fitting process.

### Statistical analysis

All statistical analyses were executed in R software (V4.4.3, https://www.R-project.org/) and SPSS27. Patients’ baseline differences between training set and testing sets were analyzed by exact Fisher test for categorical variables, A two-side hypothesis test was applied, and a p-value less than 0.05 was considered statistically significant.

The Harrell concordance index (C-index) was used for evaluating the discrimination of models. Goodness of fit (the degree of concordance between the predicted and observed values) was assessed by Calibration Curve for 2 years (730days) with 1000 bootstrap resamples using the R rms package.

Based on the prognostic score, the fixed cut-off points, the median scores of training set in three prognostic scores (T-RPS, L-RPS, TL-RPS), were used in the test sets. The different risk groups were stratified by cut-off points. The Kaplan-Meier (K-M) method was used to calculate survival rates, and log-rank test was applied to compare survival distribution between different risk groups.

## Results

### Patient characteristics

Patients baseline characteristics are reported in Table 1 and a comparative analysis was conducted on the baseline characteristics among the three data sets. The median age of the training set, test-set-1 and test-set-2 was 61.5 years (range from 21 to 88 years), 53 years (range from 44 to 75 years) and 65 years (range from 39 to 82 years), respectively. In the whole cohort, a total of 433 patients reached the endpoint. Median follow-up time was 23.4 months (range of 1.4–85.8months). More details are listed in Supplementary Table S1.

**Table 1:** Patient characteristics

Characteristic	All patients *N* = 661	Training set *N* = 292	Test-set = 1 *N* = 57	Test-set-2 *N* = 83	Test-set-3 *N* = 229	*P*-value
**Gender**						< 0.001*
Male	482(72.9%)	232(79.5%)	47(82.5%)	50(60.2%)	153(66.8%)	
Female	179(27.1%)	60(20.5%)	10(17.5%)	33(39.8%)	76(33.2%)	
**Age**						< 0.001*
≤ 60	257(38.9%)	135(46.2%)	41(71.9%)	27(32.5%)	54(23.6%)	
>60	400(60.5%)	157(53.8%)	16(28.1%)	56(67.5%)	171(74.7%)	
NA	4(0.6%)	0(0%)	0(0%)	0(0%)	4(1.7%)	
**Histology**						< 0.001*
SCC	336(50.8%)	159(54.5%)	41(71.9%)	30(36.1%)	106(46.3%)	
Non-SCC	325(49.2%)	133(45.5%)	16(28.1%)	53(63.9%)	123(53.7%)	
**Clinical Stage**						< 0.001*
IIIA	269(40.7%)	95(32.5%)	19(33.3%)	41(49.4%)	114(49.8%)	
IIIB	284(43.0%)	135(46.2%)	30(52.6%)	32(38.6%)	87(38.0%)	
IIIC	108(16.3%)	62(21.2%)	8(14.0%)	10(12.0%)	28(12.2%)	
**Tumor** **location**						0.039*
Central	286(66.2%)	189(64.7%)	46(80.7%)	51(61.4%)	——	
Peripheral	146(33.8%)	103(35.3%)	11(19.3%)	32(38.6%)	——	
**Smoking**						0.001*
No	74(18.2%)	63(21.6%)	6(10.5%)	5(6.0%)	——	
Yes	358(81.8%)	229(78.4%)	51(89.5%)	78(94.0%)	——	
**CCRT**						< 0.001*
No	236(54.6%)	194(66.4%)	42(73.7%)	0(0%)	——	
Yes	196(45.4%)	98(33.6%)	15(26.3%)	83(100%)	——	

Abbreviations NA = Null value; SCC = squamous cell carcinoma; CCRT = concurrent chemoradiotherapy

*The differences in characteristics were evaluated by exact Fisher test for categorical variables

### Radiomic feature selection and construction of models

After feature selection, a tumor-based radiomics signature including 11 radiomic features and a lung-based radiomics signature including 8 radiomic features were constructed. More details and graphs of radiomic features selection together with the selected features for model building are provided in Supplementary Materials D.

The T-RPS and the L-RPS were calculated based on the coefficients weighted by COX. Similarly, TL-RPS were also calculated. The formulae for the construction of T-RPS, L-RPS, TL-RPS are provided in Supplementary Materials E. In the integrated model (TL-RM), the weights of the T-RPS, L-RPS was 0.801897 and 0.81187, respectively.

### Performance of the models

The discrimination results are shown in Table 2. The integrated model TL-RM, which combined T-RPS and L-RPS, showed the best prediction power. The results of the tumor-based model (T-RM) and the lung-based model (L-RM) are similar (C-index 0.68 vs. 0.69 in the training set, 0.66 vs. 0.68 in the test-set-1, 0.62 vs. 0.61 in the test-set-2, 0.64 vs. 0.65 in the test-set-3). The calibration curve of 2 years OS with 1000 bootstrap resampling in training set is displayed in Supplementary Fig. S3, which shows good consistency between the predicted probabilities of 2 years OS versus the actual observed probabilities of 2 years OS.

**Table 2:** C-index of all models in different sets

Model	Training set	Test-set-1	Test-set-2	Test-set-3
	C-index (95%CI)	C-index (95%CI)	C-index (95%CI)	C-index (95%CI)
T-RM	0.68 (0.63–0.72)	0.66 (0.60–0.73)	0.62 (0.54–0.78)	0.64 (0.59–0.68)
L-RM	0.69 (0.64–0.73)	0.68 (0.62–0.75)	0.61 (0.48–0.74)	0.65 (0.55–0.74)
TL-RM	0.71 (0.66–0.77)	0.70 (0.68–0.82)	0.69 (0.58–0.84)	0.68 (0.62–0.76)

Abbreviations T-RM: COX proportional hazards model based on tumor signature; L-RM: COX proportional hazards model based on lung signature; TL-RM: COX proportional hazards model based on tumor signature and lung signature

**Fig. 2:**
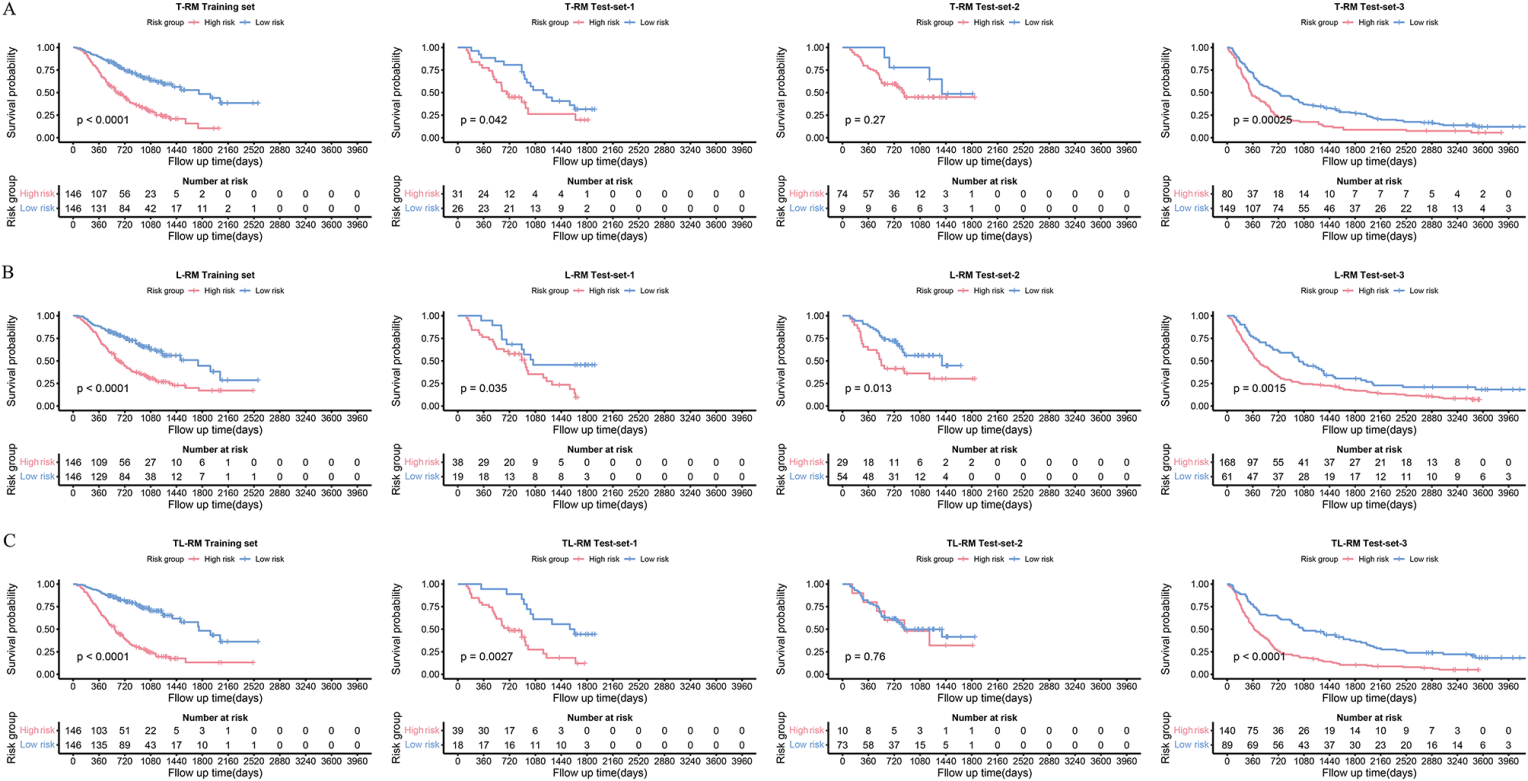
Kaplan-Meier survival curves for the tumor radiomics model (**A**), lung radiomics model (**B**), and combined tumor and lung radiomics model (**C**) in the training set and three distinct test sets (test-set-1, test-set-2, and test-set-3)

The Kaplan-Meier survival curves of T-RM, L-RM and TL-RM are provided in Fig. 2. The fixed cut-off points (the median scores of the training set) for T-RM, L-RM and TL-RM were − 321.21, -52.96 and − 300.66, respectively. The patients were divided into high-risk group and low-risk group based on the models. As shown in Fig. 2, good stratification was observed in the T-RM, L-RM and TL-RM in the training set (log-rank test, T-RM: *p* < 0.001, L-RM: *p* < 0.001, TL-RM: *p* < 0.001) and test-set-3 (log-rank test, T-RM: *p* < 0.001, L-RM: *p* = 0.0015, TL-RM: *p* < 0.001). In test-set-1, T-RM (log-rank test, *p* = 0.042), L-RM (log-rank test, *p* = 0.035) and TL-RM (log-rank test, *p* = 0.0027) showed significant stratification. In test-set-2, while the differences between the high-risk and low-risk groups in the T-RM (log-rank test, *p* = 0.27) and TL-RM (log-rank test, *p* = 0.76) were not statistically significant, the stratification of the L-RM showed a significant difference (log-rank test, *p* = 0.013) (Fig. 2). When cutoff points were determined based on the optimal cutoffs of the training set, good stratification was observed in TL-RM (log-rank test, *p* = 0.012), (Supplementary Fig. S4).

As shown in Supplementary Table S4, the tumor location, and T stage were different between the high- and low-risk groups that stratified by T-RM. For the different risk groups based on L-RM, N stage and smoking showed statistical significance (Supplementary Table S4).

## Discussion

Identification of patients with a poor prognosis and short OS is a vital clinical need on the basis of which appropriate treatment strategies can be adopted. In this study, we demonstrated that both tumor-based and lung-based radiomic features can predict OS of LA-NSCLC patients receiving definitive radiotherapy. In addition, the integrated model, combining tumor-based and lung-based radiomics signatures, can achieve an even higher predictive power.

Compared to the previous study [28], we employed a more rigorous and transparent method to select radiomic features and test the whole-lung radiomics model across three independent datasets. We believe that our approach enhances the reproducibility and generalizability of our findings. To explore the predictive power of lung radiomic features, the lung radiomics model was built and validated in three independent test sets, and patients were stratified into different risk group based on L-RM. This study showed that the predictive power of lung-based radiomics signature was similar to tumor-based radiomics signature in terms of C-index and risk group stratification. This may suggest to us that the predictive ability of latent information in the lungs is comparable to that of tumors. Moreover, according to the coefficients in tumor and lung signature combination model (TL-RSM), the importance of lung- and tumor-based radiomics signature was very close (0.81 vs. 0.80), which reminds us that lung radiomic features should not be neglected when predicting OS in LA-NSCLC.

We found that L-RM retains a good discrimination power across each of the training and test data sets (Fig. 2B). However, the stratification of T-RM and TL-R in test-set-2 performed poorly (Fig. 2A.2 C). This might be due to the fact that fewer patients were included in test-set-2. Also, this is a multi-institution North-American trial data set that might show greater heterogeneity in patient and data acquisition characteristics (i.e., patients are from different institutions). Also the lung cancer susceptibility genes and genomes are thought to exhibit variations between Chinese and Caucasian populations [29]. The training set consisted solely of Chinese patients, while the test-set-2 dataset included 84.3% Caucasian patients (Supplementary Table S1b). Even though, test-set-3 also consisted of almost exclusively Caucasian patients, it performed well perhaps because of relatively large sample size, while test-set-1 performed well perhaps because of it originated from the same data sources of training set. In addition, although we performed preprocessing such as resampling and normalization before extracting radiomic features to harmonize different datasets, there may still be image level differences due to scanning protocols, construction kernels, etc. Further in-depth algorithms still need to be developed.

Other studies have demonstrated that pre- and post-radiotherapy CT images of both have predictive ability for OS in LA-NSCLC [30–35]. In these studies, the radiomic features were derived from the primary tumor, while lung radiomic features may change after radiotherapy [36]. The main objective of this study was to explore the corrections between lung radiomic features and OS, so only pre-radiotherapy CT images were used in this study. In dataset division, selecting patients from January to December 2017 as test-set-1 minimizes end-data extreme value bias, ensures adequate follow-up, and prevents overfitting. Moreover, this period represents the IMRT to VMAT transition in the center, enhancing model generalization assessment.

Several steps were performed in this study to improve the robustness and reproducibility of the radiomic features. First, all CT images were reconstructed to the same slice thickness (1 mm), and segmentation was performed in the reconstructed images. Secondly, in terms of segmentation, manual-segmentation is considered time consuming, unstable and poorly repeatable [37]. In this study, a combination of automatic and manual segmentation method was applied to segment the lungs. Lastly, LASSO-COX was used for the selection of the radiomic features, which was suitable for high-dimensional radiomic features to reduce the effects of overfitting in small-sized data, and 1000 bootstrap method was used to reduce the risk of over-optimistic results.

Among the features selected for the tumor radiomics signature, nine radiomic features were wavelet-based features. The wavelet filtered radiomic features play a key role in the tumor-based radiomic signature. Previous studies have found similar results [23, 38, 39], suggesting that wavelet-based features reflect tumor heterogeneity well and are closely related to OS in NSCLC. Chen [40] et al. established the radiomic signature for predicting OS of NSCLC, which included the same GLDM features we found in our study.

When comparing clinical characteristics between the high- and low- risk groups defined by the radiomics prognostic score, we noted that patients with centrally located tumors had high tumor-based prognostic scores and smokers had higher lung-based prognostic scores (Supplementary Table S4). The distribution of pathological types differs between central and peripheral lung cancer, and radiomics may have the potential to discriminate between these distinct subtypes [41, 42]. Moreover, even within the same pathological type, variations in clinicopathological factors exist between central and peripheral lung cancer [43, 44], which may contribute to different prognostic scores observed in these two subgroups. Weeden [45] et al. found that a specific T cells were highly enriched in smokers and there was a different inflammatory environment between the lungs of smokers and the lungs of non-smokers, which may explain the different lung-based prognostic scores between smokers and non-smokers. This suggests to us that radiomics may reflect tumor and lung conditions and thus predict OS.

Both peri-tumoral and whole-lung radiomic features exhibit prognostic value, reflecting distinct aspects of the disease. Previous studies [46–48] indicate that peri-tumoral features are associated with prognosis, likely due to tumor invasiveness and local microenvironment changes, whereas whole-lung features may reveal systemic pathological processes such as inflammation or interstitial lung disease. Future research should focus on integrating information from multiple regions to develop a comprehensive model, elucidating the interplay between local tumor characteristics and systemic lung pathology.

There are several limitations in the present study. Firstly, this study is retrospective and prospective data will be needed for future study to eliminate selection bias. Prospective studies also enable standardization of follow-up to ensure accuracy of endpoints. Secondly, most of the patients in the study were treated before the NCCN revised its guidelines based on the results of the PACIFIC trial [49], which was one of the most revolutionary updates in the treatment of LA-NSCLC, where immunotherapy was used for patients who did not progress after radiation therapy. However, immunotherapy is presently unaffordable for many patients. For these reasons, consolidation immunotherapy has not been routinely performed in China, which may have affected OS. Even in the immunotherapy era, identifying patients who are refractory to chemoradiotherapy is useful, for these patients should not be treated with this toxic combination. In the future, we need to conduct a radiomics study of OS prediction in patients treated with curative radiotherapy and consolidation immunotherapy. Thirdly, more attention is growing on the use of multi-omics, which has been proven to improve the performance of models [50]. Through analysis of genomics, radiomics may be able to be represented at the biological level. In future studies, clinical factors, dosiomics and genomics should be integrated. Fourthly, while this study has encouraging results, this is only a pilot study and further refinement is still needed to obtain better performance for clinical use.

## Conclusion

A TRIPOD type 3 prediction model was developed and validated using external validation data. The selected lung radiomic signature has the power to predict OS in patients with LA-NSCLC treated with definitive radiotherapy in combination with chemotherapy. The model combining tumor radiomic features, and lung radiomic features has the best predictive power and can stratify patients well into different risk groups.

## Electronic supplementary material

Below is the link to the electronic supplementary material.

Supplementary Material 1

## Data Availability

Research data are stored in an institutional repository and will be shared upon request to the corresponding author.
