# Supplementary material for: Whole lung radiomic features are associated with overall survival in patients with locally advanced non-small cell lung cancer treated with definitive radiotherapy
Source: Radiat Oncol. 2025 Jan 17;20:9. doi: 10.1186/s13014-025-02583-1 (PMC11742218; doi:10.1186/s13014-025-02583-1)
Supplement: Supplementary file 1 — Supplementary Material 1 [file 13014_2025_2583_MOESM1_ESM.docx]

**Supplementary Materials**

## Supplementary material A

#### Inclusion and exclusion criteria for training and testing set

Inclusion criteria: (1) Patients was confirmed NSCLC by pathology; (2) age ≥18 years and KPS ≥70; (3) Diagnosed inoperative locally advanced NSCLC (Clinical stage II-III, American Joint Committee on Cancer, 8th edition, 2017) before radiotherapy (RT), and underwent definitive RT; (4) follow-up time over 2 years. Exclusion criteria: (1) history of lung surgery for any reason before RT; (2) history of chest RT; (3) poor quality of planning CT; (4) Unacceptable dose deviation.

#### Overall survival (OS) of the RTOG0617 data and Lung1 data from Maastro

As the OS of the RTOG0617 data and Lung1 data defined as the time from the start of RT to death, we approximate modified OS by subtracting 42days from OS of the RTOG0617 data and Lung1 data to approach the starting point of OS in the training set.

Supplementary Figure 1. Flow of patient inclusion of RTOG0617 (1a) and Lung1 in Maastro (1b) for testing.


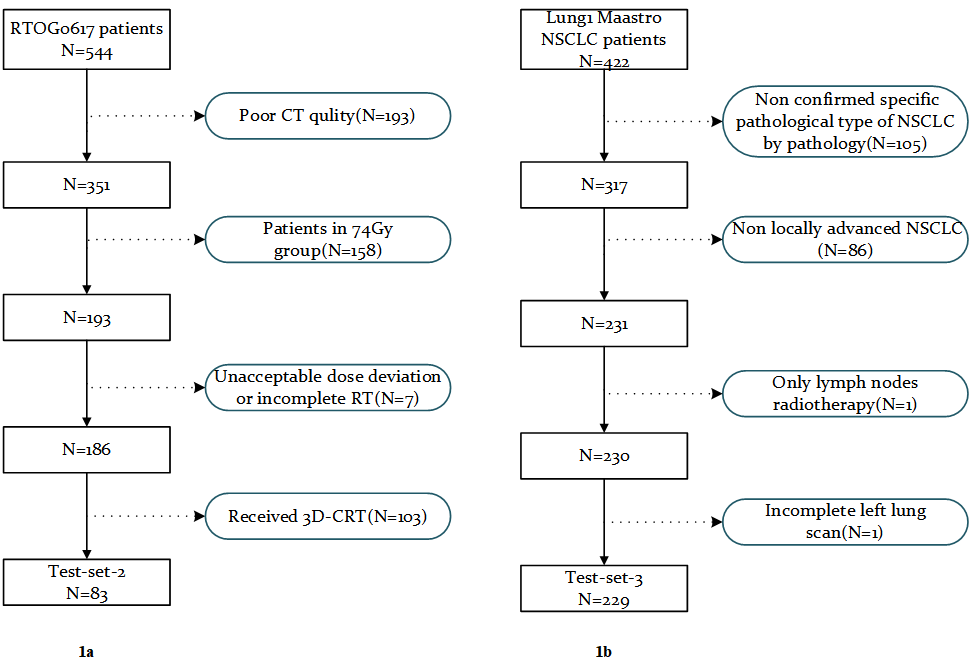


Supplementary Table 1a. Patient characteristics of Tianjin data.

|  | **All patients**  **N=349** | **Training**  **set**  **N=292** | **Test-set-1**  **N=57** | **P value** |
| --- | --- | --- | --- | --- |
| **Gender** |  |  |  | 0.604 |
| Male | 279(79.9％) | 232(79.5％) | 47(82.5％) |  |
| Female | 70(20.1％) | 60(20.5％) | 10(17.5％) |  |
| **Age** |  |  |  | <0.001* |
| ≤60 | 176(50.4％) | 135(46.2％) | 41(71.9％) |  |
| ＞60 | 173(49.6％) | 157(53.8％) | 16(28.1％) |  |
| **Histology subtype** |  |  |  | 0.015* |
| SCC | 200(57.3％) | 159(54.5％) | 41(71.9％) |  |
| Non-SCC | 149(42.7％) | 133(45.5％) | 16(28.1％) |  |
| **Location** |  |  |  | 0.019* |
| Central | 235(67.3％) | 189(64.7％) | 46(80.7％) |  |
| Peripheral | 114(32.7％) | 103(35.3％) | 11(19.3％) |  |
| **Clinical Stage** |  |  |  | 0.463 |
| IIIA | 114(32.7％) | 95(32.5％) | 19(33.3％) |  |
| IIIB | 165(47.3％) | 135(46.2％) | 30(52.6％) |  |
| IIIC | 70(20.1％) | 62(21.2％) | 8(14.0％) |  |
| **T stage** |  |  |  | 0.823 |
| T1 | 48(13.7％) | 40(13.7％) | 8(14.0％) |  |
| T2 | 84(24.1％) | 71(24.3％) | 13(22.8％) |  |
| T3 | 76(21.8％) | 61(20.9％) | 15(26.3％) |  |
| T4 | 141(40.4％) | 120(41.1％) | 21(36.8％) |  |
| **N stage** |  |  |  | 0.001* |
| N0 | 27(7.7％) | 26(8.9％) | 1(1.8％) |  |
| N1 | 27(7.7％) | 20(6.9％) | 7(12.3％) |  |
| N2 | 157(45.0％) | 121(41.6％) | 36(63.2％) |  |
| N3 | 137(39.3％) | 124(42.6％) | 13(22.8％) |  |
| **KPS** |  |  |  | 0.071 |
| ≤80 | 148(42.4％) | 130(44.5％) | 18(31.6％) |  |
| ＞80 | 201(57.6％) | 162(55.5％) | 39(68.4％) |  |
| **Loss Weight** |  |  |  | 0.478 |
| No | 287(82.2％) | 242(82.9％) | 45(78.9％) |  |
| Yes | 62(17.8％) | 50(17.1％) | 12(21.1％) |  |
| **Radiotherapy doze** |  |  |  | 0.004* |
| < 60 | 105(30.1％) | 97(33.2％) | 8(14.0％) |  |
| ≥60 | 244(69.9％) | 195(66.8％) | 49(86.0％) |  |
| **Induc chemo** |  |  |  | 0.076 |
| No | 43(12.3％) | 40(13.7％) | 3(5.3％) |  |
| Yes | 306(87.7％) | 252(86.3％) | 54(94.7％) |  |
| **CCRT** |  |  |  | 0.285 |
| No | 236(67.6％) | 194(66.4％) | 42(73.7％) |  |
| Yes | 113(32.4％) | 98(33.6％) | 15(26.3％) |  |
| **Conso chemo** |  |  |  | 0.357 |
| No | 220(63.0％) | 181(62.0％) | 39(68.4％) |  |
| Yes | 129(37.0％) | 111(38.0％) | 18(31.6％) |  |
| **Survival** |  |  |  | 0.059 |
| Alive | 156(44.7％) | 137(46.9％) | 19(33.3％) |  |
| Dead | 193(55.3％) | 155(53.1％) | 38(66.7％) |  |

*Abbreviations: KPS =* *Karnofsky performance score; Induc chemo = induction chemotherapy;* *CCRT = concurrent chemoradiotherapy; Conso chemo = consolidation chemotherapy.*

**The differences in characteristics were evaluated by Pearson X^2^ test or exact Fisher test for categorical variables.*

Supplementary Table 1b. Patient characteristics of test-set-2.

|  | **All patients** |  |
| --- | --- | --- |
|  | **N=83** | **%** |
| **Race** |  |  |
| Asian | 5 | 6.0 |
| Black or African American | 8 | 9.6 |
| White | 70 | 84.3 |
| **Ethnicity**  Hispanic or Latino  Not Hispanic or Latino  Unknown | 1  77  5 | 1.2  92.8  6.0 |
| **Zubrod**  0  1 | 51  32 | 61.4  38.6 |
| **Histology** |  |  |
| SCC | 30 | 36.1 |
| Adenocarcinoma | 34 | 41.0 |
| Large cell undifferentiated | 1 | 1.2 |
| Non-small cell lung cancer NOS | 18 | 21.7 |
| **Survival** |  |  |
| Alive | 43 | 51.8 |
| Dead | 40 | 48.2 |

*Abbreviations: SCC = squamous cell carcinoma; NOS = Not Otherwise Specified.*

## Supplementary material B

#### Details of segmentation of tumor and lung

Tumors and lungs were all segmented in pulmonary windows (window width 1400HU, window level -500HU). Malignant lymph nodes weren’t included in tumor ROIs. Both lungs (right and left lungs) were contoured. All inflated, fibrotic and emphysematic lungs were be contoured, small vessels extending beyond the hilar regions were included; however, tumor, collapsed lungs, hilars and trachea/main bronchus weren’t included in this structure.

## Supplementary material C

#### Radiomics features extraction parameter settings file

imageType:

Original:

binWidth: 25

LoG:

binWidth: 10

sigma: [1.0, 2.0, 3.0]

Wavelet:

binWidth: 5

featureClass:

shape:

- VoxelVolume

- Elongation

- Flatness

- LeastAxisLength

- MajorAxisLength

- Maximum2DDiameterColumn

- Maximum2DDiameterRow

- Maximum2DDiameterSlice

- Maximum3DDiameter

- MeshVolume

- MinorAxisLength

- Sphericity

- SurfaceArea

- SurfaceVolumeRatio

firstorder: # Remove Total Energy, correlated to Energy (due to resampling enabled)

- 10Percentile

- 90Percentile

- Energy

- Entropy

- InterquartileRange

- Kurtosis

- Maximum

- Mean

- MeanAbsoluteDeviation

- Median

- Minimum

- Range

- RobustMeanAbsoluteDeviation

- RootMeanSquared

- Skewness

- Uniformity

- Variance

glcm: # Disable SumAverage by specifying all other GLCM features available

- 'Autocorrelation'

- 'JointAverage'

- 'ClusterProminence'

- 'ClusterShade'

- 'ClusterTendency'

- 'Contrast'

- 'Correlation'

- 'DifferenceAverage'

- 'DifferenceEntropy'

- 'DifferenceVariance'

- 'JointEnergy'

- 'JointEntropy'

- 'Imc1'

- 'Imc2'

- 'Idm'

- 'Idmn'

- 'Id'

- 'Idn'

- 'InverseVariance'

- 'MaximumProbability'

- 'SumEntropy'

- 'SumSquares'

glrlm:

glszm:

gldm:

ngtdm:

setting:

interpolator: 'sitkBSpline'

resampledPixelSpacing: [2, 2, 2]

padDistance: 10 # Extra padding for large sigma valued LoG filtered images

resegmentRange: [-3, 3]

resegmentMode: sigma

voxelArrayShift: 1000

label: 1

Supplementary material D

*The Feature selection methods and results*

**Step1: Selection based on radiomic features frequencies.**

The radiomic features were screened using the least absolute shrinkage and selection operator (LASSO) embedded within the COX proportional hazards model (COX) with 5-fold cross-internal validation. This process was conducted on 1000 unique bootstrap samples derived from the entire training set. For each of the 1000 bootstraps, every radiomic feature was ranked based on its frequency of selection by the LASSO-COX model.

The top twenty features that were screened are displayed in **Supplementary Table 2**. These features are sorted according to the number of frequencies selected and are visually represented in **Supplementary Figure 2**.

To determine the optimal cut-off point, we considered the ratio of selected features to the number of training samples, maintaining a 1:10 ratio—one feature selected for every 10 samples. This ensured an appropriate feature-to-sample balance, reducing the risk of overfitting while retaining model interpretability. Consequently, we selected a cut-off point between the 10th and 20th most frequently selected features, as illustrated in Supplementary Figure 2. This range provided sufficient diversity and ensured an ample feature set for the second selection step.

**Step 2: Selection based on radiomic feature combination (signature) frequencies.**

The radiomic features selected over the cut-off point were imported into the “feature pool” for further selection based on COX and stepwise backward Akaike information criterion (AIC) for the same 1000 bootstrap samples as in step 1.

The most frequently selected radiomic feature combination (signature), ranked as the top1 in the second step of feature selection, is detailed in Supplementary Table 3. The signature of tumor includes 11 features, which were selected 9 times, while the signature of lung includes 8 features, selected 45 times.

Supplementary Table 2a. The top twenty tumor radiomics features that were selected.

| **Number** | **Radiomics Feature** | **Frequency** |
| --- | --- | --- |
| **1** | wavelet.HHH_glcm_Correlation | 790 |
| **2** | wavelet.HHL_glszm_LargeAreaLowGrayLevelEmphasis | 589 |
| **3** | wavelet.HHL_firstorder_Maximum | 557 |
| **4** | wavelet.LHL_firstorder_RootMeanSquared | 547 |
| **5** | wavelet.LLH_glcm_Correlation | 430 |
| **6** | wavelet.LLH_glcm_ClusterShade | 380 |
| **7** | wavelet.LHL_glcm_Correlation | 377 |
| **8** | original_firstorder_Skewness | 366 |
| **9** | wavelet.HLL_firstorder_Skewness | 364 |
| **10** | wavelet.HLH_glcm_Correlation | 357 |
| **11** | wavelet.LHH_firstorder_Skewness | 343 |
| **12** | wavelet.HLL_firstorder_Kurtosis | 341 |
| **13** | wavelet.LHL_glcm_ClusterShade | 341 |
| **14** | wavelet.LLL_glcm_ClusterShade | 335 |
| **15** | original_gldm_LargeDependenceHighGrayLevelEmphasis | 318 |
| **16** | wavelet.HLH_firstorder_RootMeanSquared | 304 |
| **17** | wavelet.LHL_firstorder_Kurtosis | 301 |
| **18** | wavelet.HLH_firstorder_Mean | 293 |
| **19** | wavelet.HLH_glcm_JointAverage | 282 |
| **20** | original_glszm_SmallAreaEmphasis | 270 |

Supplementary Table 2b. The top twenty lung radiomics features that were selected.

| **Number** | **Radiomics Feature** | **Frequency** |
| --- | --- | --- |
| 1 | wavelet.HLL_firstorder_Maximum | 963 |
| 2 | original_shape_Sphericity | 778 |
| 3 | original_shape_Maximum3DDiameter | 749 |
| 4 | wavelet.HLH_firstorder_Mean | 725 |
| 5 | wavelet.LHL_firstorder_Skewness | 654 |
| 6 | wavelet.HLH_glcm_Imc2 | 616 |
| 7 | wavelet.HLL_glszm_ZoneEntropy | 578 |
| 8 | original_shape_Maximum2DDiameterSlice | 503 |
| 9 | wavelet.LHH_glszm_LargeAreaHighGrayLevelEmphasis | 491 |
| 10 | original_glszm_LargeAreaHighGrayLevelEmphasis | 459 |
| 11 | wavelet.HHL_firstorder_Skewness | 424 |
| 12 | wavelet.LLH_firstorder_Mean | 423 |
| 13 | wavelet.LHL_firstorder_Maximum | 400 |
| 14 | wavelet.LLH_glszm_ZoneEntropy | 400 |
| 15 | wavelet.LLH_gldm_SmallDependenceLowGrayLevelEmphasis | 390 |
| 16 | original_firstorder_Energy | 372 |
| 17 | wavelet.HHH_firstorder_Skewness | 372 |
| 18 | wavelet.HLH_glcm_Correlation | 370 |
| 19 | original_shape_LeastAxisLength | 364 |
| 20 | original_gldm_SmallDependenceLowGrayLevelEmphasis | 346 |

Supplementary Figure 2. (a) The tumor radiomics features are sorted according to the number of frequencies selected. (b) The lung radiomics features are sorted according to the number of frequencies selected.


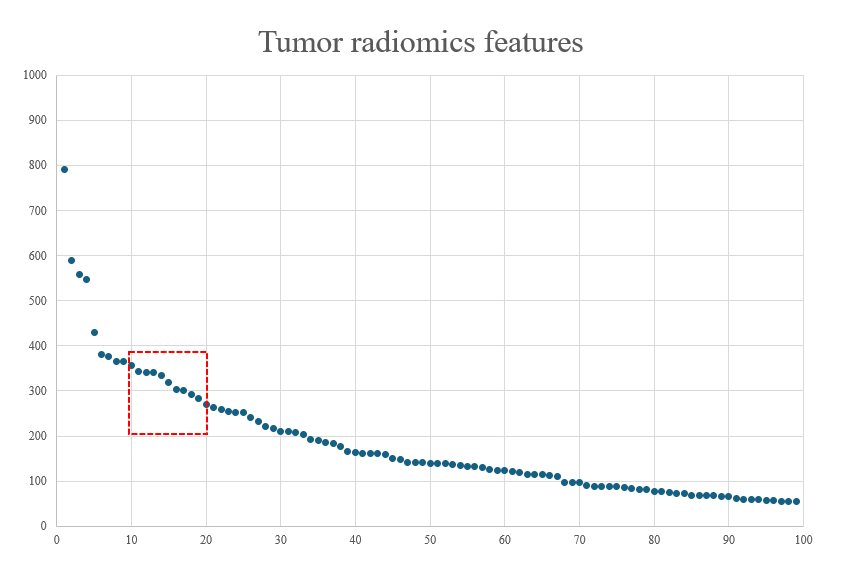


Supplementary Figure 2a


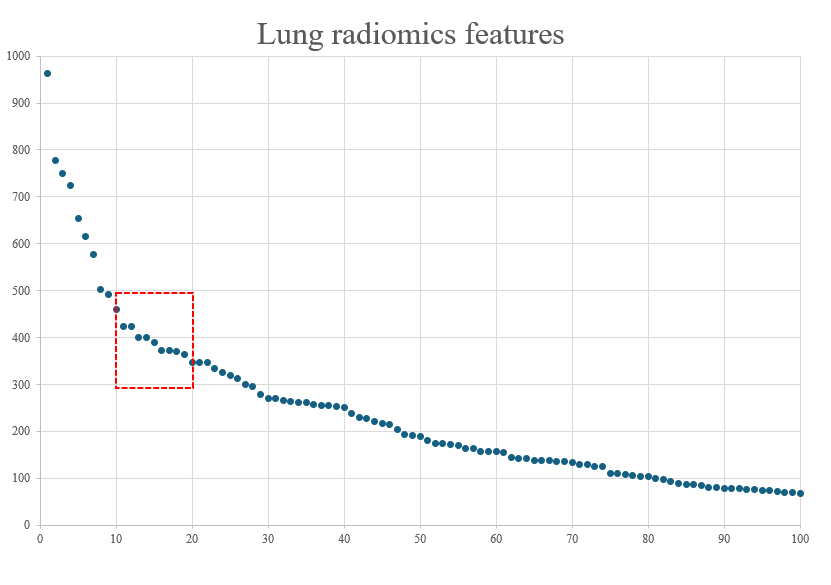


Supplementary Figure 2b

Supplementary Table 3. The top one frequently selected radiomics signature.

| **Radiomics signature** | **Radiomics features** |
| --- | --- |
| Tumor | original_firstorder_Skewness |
|  | original_gldm_LargeDependenceHighGrayLevelEmphasis |
|  | wavelet.HHH_glcm_Correlation |
|  | wavelet.HHL_firstorder_Maximum |
|  | wavelet.HHL_glszm_LargeAreaLowGrayLevelEmphasis |
|  | wavelet.HLH_firstorder_RootMeanSquared |
|  | wavelet.LHL_firstorder_Kurtosis |
|  | wavelet.LHL_firstorder_RootMeanSquared |
|  | wavelet.LLH_glcm_ClusterShade |
|  | wavelet.LHL_glcm_Correlation |
|  | wavelet.LLL_glcm_ClusterShade |
| Lung | original_glszm_LargeAreaHighGrayLevelEmphasis |
|  | original_shape_Maximum2DDiameterSlice |
|  | original_shape_Maximum3DDiameter |
|  | wavelet.HLH_firstorder_Mean |
|  | wavelet.HLL_firstorder_Maximum |
|  | wavelet.HLL_glszm_ZoneEntropy |
|  | wavelet.LHH_glszm_LargeAreaHighGrayLevelEmphasis |
|  | wavelet.LLH_firstorder_Mean |

*The formulae for the construction of T-RPS, L-RPS, TL-RPS.*

T-RPS = (-0.344822)×original_firstorder_Skewness

+(-9.840087E-06)×original_gldm_LargeDependenceHighGrayLevelEmphasis

+(-30.985651)×wavelet.HHH_glcm_Correlation

+0.001839×wavelet.HHL_firstorder_Maximum

+(-5.479932)×wavelet.HHL_glszm_LargeAreaLowGrayLevelEmphasis

+(-0.297379)×wavelet.HLH_firstorder_RootMeanSquared +0.037581×wavelet.LHL_firstorder_Kurtosis

+(-0.027175)×wavelet.LHL_firstorder_RootMeanSquared +0.000012×wavelet.LLH_glcm_ClusterShade

+3.549162×wavelet.LLH_glcm_Correlation

+(-2.578180E-08)×wavelet.LLL_glcm_ClusterShade

L-RPS = (3.779243E-08)×original_glszm_LargeAreaHighGrayLevelEmphasis

+(-0.029704)×original_shape_Maximum2DDiameterSlice

+0.037520×original_shape_Maximum3DDiameter

+2.678601×wavelet-HLH_firstorder_Mean

+(-0.002369)×wavelet-HLL_firstorder_Maximum

+ (-7.171278)×wavelet-HLL_glszm_ZoneEntropy

+(-2.029555E-09)×wavelet-LHH_glszm_LargeAreaHighGrayLevelEmphasis

+ 0.274985×wavelet-LLH_firstorder_Mean

TL-RPS = 0.801897×T-RPS+0.811871×L-RPS.

## Supplementary material E

Supplementary Figure 3. The calibration curve of 2 years OS in training set.


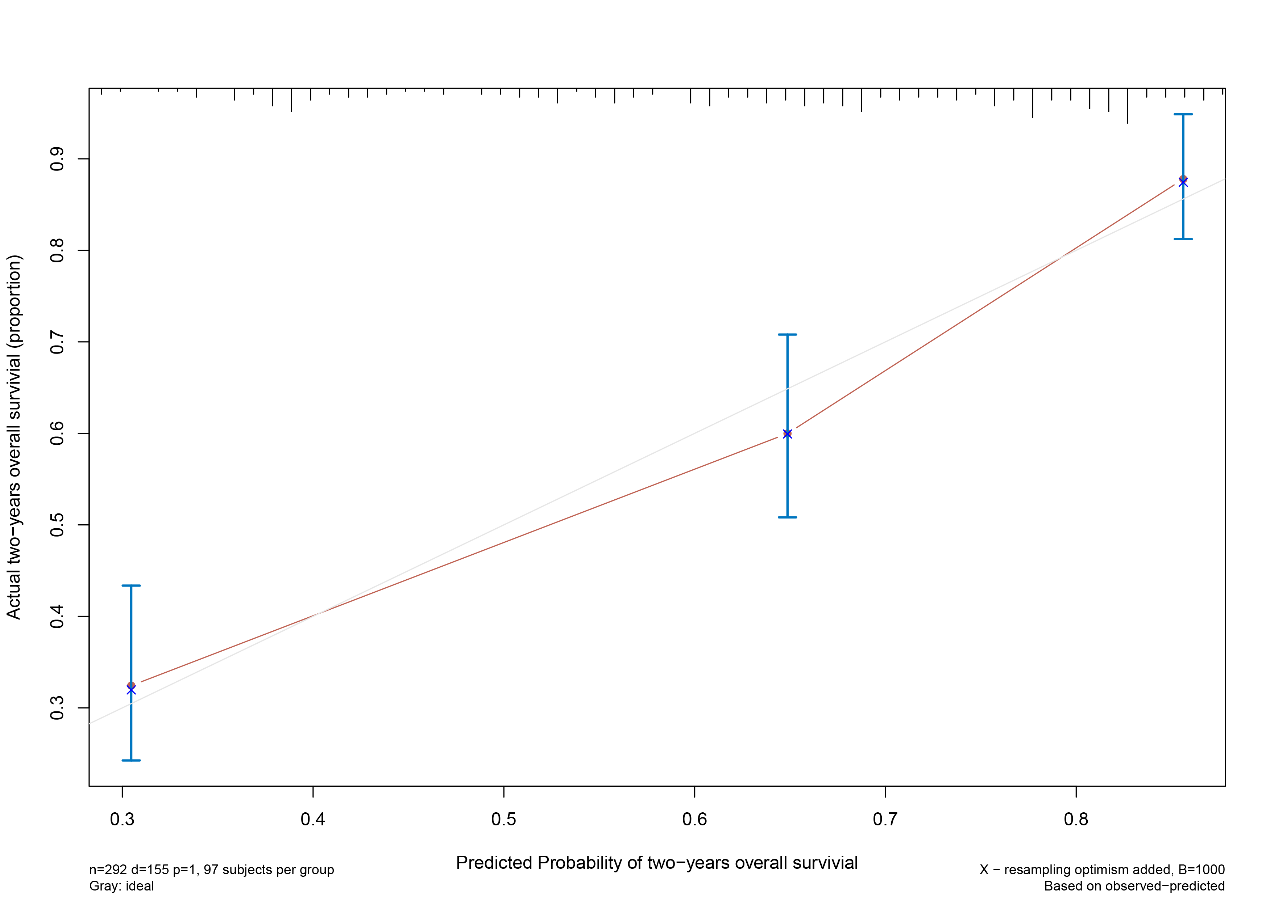


## Supplementary material F

Supplementary Table 4a1. Distribution of clinical factors in different risk groups, based on T-RM in whole cohort, training set and test sets (optimal cutoff point of training set).

|  | **Whole cohort** | | | **Training set** | | | **Test sets** | | |
| --- | --- | --- | --- | --- | --- | --- | --- | --- | --- |
| **Clinical**  **factors** | **High-risk**  **N=271** | **Low-risk**  **N=161** | **P-value** | **High-risk**  **N=204** | **Low-risk**  **N=88** | **P-value** | **High-risk**  **N=67** | **Low-risk**  **N=73** | P-value |
| **Tumor**  **location** |  |  | 0.036* |  |  | 0.084 |  |  | 0.102 |
| Central | 189(69.7％) | 96(59.6％) |  | 138(67.6％) | 50(56.8％) |  | 51(76.1％) | 46(63.0％) |  |
| Peripheral | 82(30.3％) | 65(40.4％) |  | 66(32.4％) | 38(43.2％) |  | 16(23.9％) | 27(37.0％) |  |
| **T Stage** |  |  | <0.001* |  |  | 0.032 |  |  | <0.001* |
| 1 | 25(9.2％) | 41(25.5％) |  | 20(9.8％) | 20(22.7％) |  | 5(7.5％) | 21(28.8％) |  |
| 2 | 66(24.4％) | 43(26.7％) |  | 51(25.0％) | 20(22.7％) |  | 15(22.4％) | 23(31.5％) |  |
| 3 | 66(24.4％) | 32(19.9％) |  | 47(23.0％) | 14(15.9％) |  | 19(28.4％) | 18(24.7％) |  |
| 4 | 114(42.1％) | 45(28.0％) |  | 86(42.2％) | 34(38.6％) |  | 28(41.8％) | 11(15.1％) |  |

*Abbreviations: T-RM: COX regression model based on tumor signature.*

**The differences in characteristics were evaluated by Pearson X^2^ test or exact Fisher test for categorical variables.*

Supplementary Table 4a2. Distribution of clinical factors in different risk groups, based on T-RM in whole cohort, training set and test sets (cutpoint= median score of training set).

|  | **Whole cohort** | | | **Training set** | | | **Test sets** | | |
| --- | --- | --- | --- | --- | --- | --- | --- | --- | --- |
| **Clinical**  **factors** | **High-risk**  **N=251** | **Low-risk**  **N=181** | **P-value** | **High-risk**  **N=146** | **Low-risk**  **N=146** | **P-value** | **High-risk**  **N=105** | **Low-risk**  **N=35** | **P-value** |
| **Tumor**  **location** |  |  | 0.471 |  |  | 0.086 |  |  | 0.057 |
| Central | 170(67.7％) | 116(64.1％) |  | 102(69.9％) | 87(59.6％) |  | 68(64.8％) | 29(82.9％) |  |
| Peripheral | 81(32.3％) | 65(35.9％) |  | 44(30.1％) | 59(40.4％) |  | 37(25.2％) | 6(17.1％) |  |
| **T Stage** |  |  | 0.446 |  |  | 0.102 |  |  | 0.832 |
| 1 | 35(13.9％) | 31(17.1％) |  | 14(9.6％) | 26(17.8％) |  | 21(20.0％) | 5(14.3％) |  |
| 2 | 60(23.9％) | 49(27.1％) |  | 33(22.6％) | 38(26.0％) |  | 27(25.7％) | 11(31.4％) |  |
| 3 | 63(25.1％) | 35(19.3％) |  | 36(24.7％) | 25(17.1％) |  | 27(25.7％) | 10(28.6％) |  |
| 4 | 93(37.1％) | 66(36.5％) |  | 63(43.2％) | 57(39.0％) |  | 30(28.6％) | 9(25.7％) |  |

*Abbreviations: T-RM: COX regression model based on tumor signature.*

**The differences in characteristics were evaluated by Pearson X^2^ test or exact Fisher test for categorical variables.*

Supplementary Table 4b1. Distribution of clinical factors in different risk groups, based on L-RM in whole cohort, training set and test sets (optimal cutoff point of training set).

|  | **Whole cohort** | | | **Training set** | | | **Test sets** | | |
| --- | --- | --- | --- | --- | --- | --- | --- | --- | --- |
| **Clinical**  **factors** | **High-risk**  **N=173** | **Low-risk**  **N=259** | **P-value** | **High-risk**  **N=111** | **Low-risk**  **N=181** | **P-value** | **High-risk**  **N=62** | **Low-risk**  **N=78** | **P-value** |
| **N Stage** |  |  | 0.024* |  |  | 0.015* |  |  | 0.906 |
| 0 | 8(4.6％) | 19(7.3％) |  | 8(7.2％) | 18(9.9％) |  | 0(0.0％) | 1(1.3％) |  |
| 1 | 7(4.0％) | 26(10.0％) |  | 2(1.8％) | 18(9.9％) |  | 5(8.1％) | 8(10.3％) |  |
| 2 | 98(56.6％) | 116(44.8％) |  | 55(49.5％) | 67(37.0％) |  | 43(69.4％) | 49(62.8％) |  |
| 3 | 60(34.7％) | 98(37.8％) |  | 46(41.4％) | 78(43.1％) |  | 14(22.6％) | 20(25.6％) |  |
| **Smoking** |  |  | <0.001* |  |  | 0.003* |  |  | 0.023* |
| No | 15(8.7％) | 59(22.8％) |  | 14(12.6％) | 49(27.1％) |  | 1(1.6％) | 10(12.8％) |  |
| Yes | 158(91.3％) | 200(77.2％) |  | 97(87.4％) | 132(72.9％) |  | 61(98.4％) | 68(87.2％) |  |

*Abbreviations:* L-RM: COX regression model based on lung signature.

**The differences in characteristics were evaluated by Pearson X^2^ test or exact Fisher test for categorical variables.*

Supplementary Table 4b2. Distribution of clinical factors in different risk groups, based on L-RM in whole cohort, training set and test sets (cutpoint= median score of training set).

|  | **Whole cohort** | | | **Training set** | | | **Test sets** | | |
| --- | --- | --- | --- | --- | --- | --- | --- | --- | --- |
| **Clinical**  **factors** | **High-risk**  **N=213** | **Low-risk**  **N=219** | **P-value** | **High-risk**  **N=146** | **Low-risk**  **N=146** | **P-value** | **High-risk**  **N=67** | **Low-risk**  **N=73** | **P-value** |
| **N Stage** |  |  | 0.159 |  |  | 0.151 |  |  | 0.371 |
| 0 | 11(5.2％) | 16(7.3％) |  | 10(6.8％) | 16(11.0％) |  | 1(1.5％) | 0(0.0％) |  |
| 1 | 11(5.2％) | 22(10.0％) |  | 7(4.8％) | 13(8.9％) |  | 4(6.0％) | 9(12.3％) |  |
| 2 | 113(53.1％) | 101(46.1％) |  | 69(47.3％) | 53(36.3％) |  | 44(65.7％) | 48(65.8％) |  |
| 3 | 78(36.6％) | 80(36.5％) |  | 60(41.1％) | 64(43.8％) |  | 18(26.9％) | 16(21.9％) |  |
| **Smoking** |  |  | <0.001* |  |  | 0.010* |  |  | 0.010* |
| No | 23(10.8％) | 51(23.3％) |  | 22(15.1％) | 41(28.1％) |  | 1(1.5％) | 10(13.7％) |  |
| Yes | 190(89.2％) | 168(76.7％) |  | 124(84.9％) | 105(71.9％) |  | 66(98.5％) | 63(86.3％) |  |

*Abbreviations:* L-RM: COX regression model based on lung signature.

**The differences in characteristics were evaluated by Pearson X^2^ test or exact Fisher test for categorical variables.*

Supplementary material G


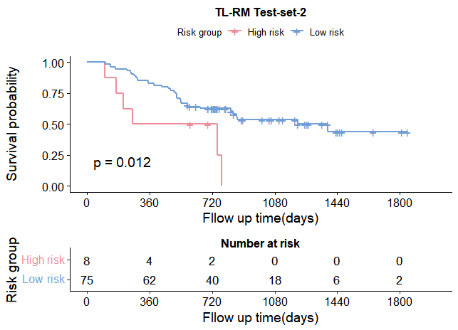


Supplementary Figure4. Kaplan-Meier survival curves based on the TL-RM according to the optimal cutoff point of training set in test-set-2.*Abbreviations:* TL-RM: COX proportional hazards model based on tumor signature and lung signature.
